# Genetic Parameters and Genome‐Wide Association Studies for Fertility and Reproduction Traits in U.S. Katahdin Sheep Based on the Single‐Step GBLUP Methodology

**DOI:** 10.1111/jbg.70048

**Published:** 2026-03-30

**Authors:** Alejandra Toro Ospina, Ronald M. Lewis, Bradley A. Freking, Joan M. Burke, Thomas W. Murphy, Carrie S. Wilson, Luiz F. Brito

**Affiliations:** ^1^ Department of Animal Sciences Purdue University West Lafayette Indiana USA; ^2^ Department of Animal Science University of Nebraska‐Lincoln Lincoln Nebraska USA; ^3^ USDA‐ARS, U.S. Meat Animal Research Center Clay Center Nebraska USA; ^4^ USDA‐ARS, Dale Bumpers Small Farms Research Center Booneville Arkansas USA; ^5^ USDA‐ARS, Range Sheep Production Efficiency Research Unit Dubois Idaho USA

**Keywords:** fertility, GWAS, hair sheep, reproductive performance, small ruminants

## Abstract

Sheep production contributes to a secure and diverse food and fibre supply in the United States, with growing ethnic diversity strengthening demand. Katahdin is a composite hair‐type sheep breed developed in the United States that has become the most popular breed in many regions of the country and the first one to have genomic selection implemented in its breeding program. Therefore, the main objectives of this study were to estimate variance components of reproductive traits, including number of lambs born (NLB), number of lambs weaned (NLW), age at first lambing (AFL), and interval from first to second lambing (LI), in Katahdin sheep using the AIREML method and the single‐step Genomic Best Linear Unbiased Prediction (ssGBLUP) approach, and to identify genomic regions and candidate genes associated with these traits. The datasets used consisted of 127,536 animals in the pedigree, phenotypic records of 56,128 parities from 24,067 ewes, and genomic data from 10,032 animals with 30,308 single‐nucleotide polymorphisms (SNP) after quality control. Analyses were performed using the BLUPF90 family of programs. We observed low heritability estimates for all studied traits (0.09 ± 0.00 for NLB, 0.08 ± 0.00 for NLW, 0.09 ± 0.01 for AFL, and 0.08 ± 0.01 for LI). The genetic correlations between the traits ranged from 0.17 ± 0.02 (AFL and LI) to 0.79 ± 0.02 (NLB and NLW). All traits were found to be highly polygenic with all 14 significant SNP on eight (OAR) chromosomes (3, 6, 7, 8, 9, 12, 13, and 15) having small effects on the total variability on the traits. These SNP were located near or within 18 candidate genes: four genes associated with NLB (*AAK1, GFPT1, SLC23A2,* and *GDAP1*), four with NLW (*ARHGAP18, TTLL2, UNC93A,* and *GPR31*), six with AFL (*NAP1L5, FAM13A, HS3ST1, CCDC181, NME7,* and *BLZF1*), and four with LI (*TAF4, CDH4, CADM1*, and *SEL1L*). These candidate genes have been previously associated with fertility, embryonic development, growth, disease resistance, and climatic adaptation traits. Our findings indicate that fertility and reproduction traits in Katahdin sheep can be improved through direct genetic selection. Genetic improvement for these traits will benefit from genomic selection as more accurate estimates of breeding values for selection candidates can be obtained at a younger age. Although the studied traits are influenced by a complex interplay of genetic and environmental factors, the candidate genes identified enabled a better understanding of the biological mechanisms underlying reproductive performance in Katahdin sheep.

## 1 Introduction

Sheep production is an essential part of the global livestock industry, providing income through the production of meat, milk, wool, and skin (Ajafar et al. 2022; Aydin et al. 2024). Katahdin is a composite hair sheep breed valued for its climatic adaptability, productivity, maternal ability, and reproductive efficiency. Katahdin is one of the fastest‐growing sheep breeds in the United States and the first to have genomic selection implemented in its national breeding program. Two reproductive performance traits are included in the Katahdin genetic evaluation: number of lambs born (NLB) as an indicator of ewe prolificacy, and number of lambs weaned (NLW) as an indicator of ewe rearing ability and lamb viability (Notter et al. 2018).

Reproductive traits are complex, with underlying biological mechanisms related to fertility, animal development, and physiological indicators of reproduction (e.g., ovulation rate, age at first lambing, lambing interval (LI), litter size, body weight, and lamb survival rates; Abdoli et al. 2019a, 2019b). Fertility and reproduction traits are generally lowly heritable and highly polygenic in both cattle (Fonseca et al. 2018; Alves et al. 2020) and sheep (Bolormaa et al. 2017; Abdoli et al. 2019a, 2019b; Tera et al. 2021). However, their genomic background in Katahdin and other hair sheep breeds has been less studied, possibly due to the limited availability of genomic information and phenotypic records on commercial farms (Hernandez et al. 2011).

Important reproductive traits, such as age at first lambing (AFL), an indicator of ewe precocity, and LI, are not commonly evaluated in sheep breeding programs. These traits are important because they are directly related to flock profitability (Vanimisetti and Notter 2012; Pinto et al. 2024). Two studies have reported low heritability estimates for NLB and NLW in Katahdin sheep (Vanimisetti et al. 2007; Notter et al. 2018), but no studies have evaluated other reproductive traits in this breed. Low heritability estimates for reproductive traits have also been reported in other sheep breeds (Abdoli et al. 2019a, 2019b; Araujo et al. 2023), reinforcing their genetic complexity and polygenicity. Furthermore, reproduction traits are strongly affected by environmental factors and are typically expressed later in life, making it more difficult to achieve rapid genetic progress in sheep populations.

Genomic selection is a powerful tool for increasing genetic gain for lowly heritable traits that are sex‐limited or expressed late in the life of the animals (Goddard and Hayes 2007; Meuwissen et al. 2016). Genomic selection has become a common practice in various livestock breeding programs, including those for dairy cattle (Weller et al. 2017; Wiggans and Carrillo 2022), beef cattle (Zhang et al. 2013; Magalhães et al. 2019), pigs (Samorè and Fontanesi 2016), and poultry (Fulton 2012). However, the application of genomic tools in sheep breeding has been more limited until recently. Even with a small number of genotyped animals, the single‐step Genomic Best Linear Unbiased Prediction (ssGBLUP) method (Legarra et al. 2009; Christensen and Lund 2010) can be used for integrating data from genotyped and non‐genotyped animals into a single analysis when computing genomically enhanced breeding values (GEBV; Legarra et al. 2009). Furthermore, the ssGBLUP approach can be extended to perform single‐step genome‐wide association studies (ssGWAS; Wang et al. 2014) by back‐solving GEBV and calculating approximate *p*‐values (Aguilar et al. 2019). ssGWAS enable the identification of genetic markers and genomic regions statistically associated with traits of interest (Rodrigues et al. 2024; Carvalho et al. 2020).

Several studies have used genomic information in sheep to estimate genetic parameters (Pinto et al. 2025a, 2025b; Kasap et al. 2021), predict GEBV (Brito et al. 2017; Hernández‐Montiel et al. 2020; Jiang et al. 2021; Araujo et al. 2023; McMillan et al. 2022), and conduct GWAS for traits such as gastrointestinal parasite resistance (Notter et al. 2022; Becker et al. 2022), body weight (Rovadoscki et al. 2018), longevity indicators (Pinto et al. 2025a, 2025b), and wool production (Bolormaa et al. 2017). Despite the increasing popularity of the Katahdin breed in the United States, which now has the highest percentage of registered ewes (26.9%) in the National Sheep Improvement Program (NSIP; Nilson et al. 2024), genomic studies in the breed (and in sheep in general) remain limited due to the high cost of genotyping relative to the value of each animal and breeding program costs.

No GWAS studies have been reported on growth traits (e.g., birth weight, maternal weaning weight, or post‐weaning weight) or reproductive traits (e.g., NLB, NLW) in the Katahdin breed. There are also limited reports of genomic studies or estimates of genetic parameters for reproductive traits in this breed, even though such traits are key to sustainable sheep production. Therefore, the main objectives of this study were to: (1) estimate variance components and genetic parameters for NLB, NLW, AFL, and LI in Katahdin sheep based on the ssGBLUP approach; and, (2) perform ssGWAS for these four traits to identify genomic regions and candidate genes associated with reproduction, and to enhance the understanding of the biological mechanisms underlying the phenotypic expression of these traits in Katahdin sheep.

## 2 Material and Methods

Approval from the Animal Use and Ethics Committee was not needed for this study as preexisting datasets were provided by the NSIP and recorded by U.S. Katahdin producers during routine husbandry activities.

### 2.1 Phenotypic and Genomic Datasets

This study used phenotypic records provided by Katahdin sheep breeders and made available through the NSIP (www.nsip.org). Data editing and calculation of descriptive statistics for the four traits analysed, that is, NLB and NLW recorded for each parity of the ewes, AFL, and LI, were performed using the R packages ‘dplyr’ (Wickham et al. 2023) and ‘lubridate’ (Grolemund and Wickham 2011).

A total of 26,192 ewes with 66,374 phenotypic records (1997–2023) for NLB and NLW from 200 flocks were used. Table S1 presents the distribution of the number of records per ewe for NLB and NLW. Since AFL and LI traits are not directly recorded nor currently included in the breeding program, they were derived from existing information. AFL was calculated as the difference in days between the date of the first lambing and the animal's birth date. Records with AFL exceeding 60 months (> 5 years) were removed as they most likely reflect recording errors. LI was calculated as the difference in days between the dates of first and second lambing. For LI, only the interval between the first and second lambings was used, as this information was the most complete and reliable in the dataset. Later records showed greater variability and inconsistency, possibly due to unrecorded lambings or seasonal reproductive management practices. Additionally, phenotypic records that deviated by more than three standard deviations from the mean were excluded from subsequent analyses.

Genotypic information from 10,032 animals with 32,738 SNPs was provided by NSIP. Quality control of the genotype data was performed using the PreGSF90 package (Aguilar et al. 2010). We removed SNPs with a call rate lower than 0.90, minor allele frequency (MAF) lower than 0.05, SNPs with extreme departure from the Hardy Weinberg Equilibrium based on an excess of heterozygosity greater than 0.15, monomorphic SNPs, SNPs with unknown or duplicated positions, and SNPs located on non‐autosomal chromosomes. We also removed animals with a call rate lower than 0.90. After the quality control, 30,885 SNPs and 10,032 animals remained for subsequent analyses.

### 2.2 Pedigree Information and Parent‐Offspring Mismatch Analyses

Pedigree information was obtained from NSIP and included 127,536 animals born between 1991 and 2023, comprising 3307 sires and 26,344 dams, with 9207 animals having missing parental information. Parentage verification was performed to detect and correct pedigree errors based on the available genomic information, using the SeekParent software version 1.56. Out of the 10,032 genotyped animals, parent‐offspring conflicts were identified in 604 cases. This included 534 instances of father‐offspring conflicts and 70 instances of mother‐offspring conflicts. Corrections to these relationships were made for 105 animals. In 449 cases, the parents could not be identified because they were not included in the genomic database. Animals with parental conflicts were considered as having missing parent(s) in the pedigree.

#### 2.2.1 Variance Components Analyses

Two single‐trait models were employed for the analyses. Model (1) was used to analyse AFL and LI while Model (2) was applied to NLB and NLW. The effects included in each model were adjusted according to the specific characteristics of the trait being analysed. In both models, contemporary group (CG; defined by flock, year of birth, lambing number, season, and management group) and age of ewe (ewes older than 7 years were included in the same group) were included as fixed effects. CG with fewer than three animals was excluded from the analyses. Furthermore, for NLB and NLW, the random permanent environment effect was fitted to account for the repeated records on an animal. These two models were defined as:

(Model 1) $$y_{1}=\mathrm{Xb}+Z_{1}a+e$$

(Model 2) $$y_{2}=\mathrm{Xb}+Z_{1}a+Z_{2}c+e$$

where **y** is the vector of phenotypic observations; **b** is the vector of fixed effect solutions, which included CG and age of the ewe; **a** is a vector of random direct additive genetic effects; 𝒄 is a vector of permanent environmental effects (included only in Model (2)), and 𝒆 is a vector of random residual errors. The incidence matrices 𝑿, 𝒁_1_, and ***Z***_2_ link the phenotypic observations with the fixed, additive genetic, and permanent environmental effects, respectively. The following assumptions were considered for the random effects: $a\sim N\left(0,H\sigma_{a}^{2}\right),c\sim N\left(0,I\sigma_{\mathrm{pe}}^{2}\right)$, and $e\sim N\left(0,I\sigma_{e}^{2}\right),$ where ***H*** represents the hybrid genomic relationship matrix (Legarra et al. 2009), ***I*** is an identity matrix of appropriate order.

Variance components were estimated using the Average Information Restricted Maximum Likelihood (AIREML) method implemented in the BLUPF90+ software (Misztal et al. 2022). The two‐trait models were analysed under the ssGBLUP approach, evaluating two trait combinations, NLB–NLW and AFL–LI, using the same fixed and random effects structure as applied in the univariate analyses. Genetic correlations were derived from the estimated additive genetic (co)variance matrix. The individual accuracy of the GEBV was calculated using the formula:

$${\text{Accuracy}}_{i}=\sqrt{1-\frac{{\mathrm{PEV}}_{i}}{{\hat{\sigma }}_{a}^{2}}}$$

where PEV_*i*_ represents the prediction error variance of the GEBV for animal *i*, and $\sigma_{a}^{2}$ is the estimated additive genetic variance for the trait being analysed. Genetic trends were plotted based on annual average GEBV of animals born from 2007 to 2022. The genetic base was defined as animals born in 2007 and genetic trends were centered relative to this base year and scaled by the additive genetic standard deviation ($\hat{\sigma_{a}}$) to allow comparisons among traits. Plots were generated using the *ggplot2* R package (Tollefson 2021).

#### 2.2.2 GWAS Analyses

Single‐step genome‐wide association studies were performed using the BLUPF90 and PostGSF90 programs (Misztal et al. 2022). SNP effects were obtained by back‐solving genomic breeding values using the PostGSF90 package (Aguilar et al. 2014) and the ssGWAS approach. Multiple‐testing correction was applied by adjusting the significance level (*α*) according to the Bonferroni approach. Chromosome‐wise significance thresholds were calculated as *α*_*i*_ $=\frac{0.05}{Me_{i}}$, where *α*_*i*_ is the threshold for the *i*th chromosome and Me_*i*_ represents the number of independent segregating segments on each chromosome of the sheep genome. Me_*i*_ was calculated with the ‘GALLO’ R package (Fonseca et al. 2020) using a linkage disequilibrium‐based method and considering the characteristics of the sheep reference genome assembly GCF_016772045.2 (www.ncbi.nlm.nih.gov). The genome‐wise significance threshold was determined using the formula: $\frac{0.05}{\left(\sum_{i=1}^{n}Me_{i}\right)}$, where *n* is the number of chromosomes included in the analyses, and Me_*i*_ corresponds to the number of effective independent tests per chromosome. Based on this approach, the calculated genome‐wise threshold was 2.12 × 10^−5^ and the chromosome‐wise thresholds ranged from 2.29 × 10^−4^ to 9.53 × 10^−4^. Manhattan and QQ plots were generated using the ‘qqman’ R package (Turner 2018).

### 2.3 Functional Annotation Analyses

To annotate the significant regions in the GWAS analyses, the GALLO R package version 1.5 (Fonseca et al. 2020) was used to identify candidate genes and QTL within a 100 Kb genomic window on each side of the significant SNP. This window was defined based on the linkage disequilibrium pattern in the breed. The genomic coordinates of the ARS‐UI_Ramb_v2.0.112 reference genome (https://ftp.ensembl.org/pub/release‐112/gtf/ovis_aries_rambouillet/) were used as input files (.gtf, Ensembl), along with the quantitative trait loci (QTL) map QTLdb_sheepOAR_rambo2.gff (SheepQTLdb, version 53). Functional annotation and metabolic pathway analyses were performed using the DAVID (Database for Annotation, Visualization, and Integrated Discovery) platform (Sherman et al. 2022).

### 2.4 Descriptive Statistics

The descriptive statistics for the four traits, following the quality control process, are presented in Table 1. NLB ranged from 1 to 3, with a mean of 1.74. NLW ranged from 0 to 3, with a slightly lower mean of 1.61. Both traits showed similar medians and quartiles, indicating consistent lamb prolificacy and survival to weaning. AFL ranged from 300 to 600 days, with a mean of 403.5 days and SD of 75.1 suggesting considerable variability in the onset of reproductive maturity. LI ranged from 183 to 707 days, with a mean of 356.4 days and SD of 131, reflecting a wide range in the time between the first two lambings. This variation is likely attributable to environmental and management effects, which influence fertility and reproductive traits.

**TABLE 1:** Descriptive statistics for the number of lambs born (NLB), number of lambs weaned (NLW), age at first lambing (AFL), and interval between the first and second lambings (LI) in Katahdin sheep.

Trait	Number of individuals	Number of records	Minimum	Mean	Maximum
NLB (count)	24,067	56,128	1	1.74	3
NLW (count)	24,067	56,128	0	1.61	3
AFL (days)	13,409	13,409	300	403.50	600
LI (days)	13,409	13,409	183	356.40	707

## 3 Results

### 3.1 Variance Components and Genetic Parameters

The estimates of variance components and genetic parameters for each trait are presented in Table 2. Overall, the residual variance represented a substantial proportion of the total phenotypic variance, reflecting the impact of both environmental effects and unmodeled genetic components, such as dominance and epistasis, on the expression of the studied traits. In general, all traits were lowly heritable, indicating that the environment had a strong influence on their phenotypic expression. Repeatability estimates for NLB and NLW indicate the value of repeated records for increasing the accuracy of (G)EBV. The genetic correlation between NLB and NLW was 0.79 ± 0.02, supporting a common genetic basis between the two traits. In contrast, the genetic correlation between AFL and LI was low (0.17 ± 0.02).

**TABLE 2:** Estimates of variance components for number of lambs born (NLB), number of lambs weaned (NLW), age at first lambing (AFL), and interval between first and second lambings (LI) in Katahdin sheep.

Trait	$\sigma_{a}^{2}$	$\sigma_{\mathrm{pe}}^{2}$	$\sigma_{e}^{2}$	$\sigma_{p}^{2}$	*h* ^2^	*R*
NLB	0.04 ± 0.00	0.03 ± 0.00	0.34 ± 0.00	0.41 ± 0.04	0.09 ± 0.00	0.18 ± 0.00
NLW	0.03 ± 0.00	0.03 ± 0.00	0.35 ± 0.00	0.42 ± 0.02	0.08 ± 0.00	0.16 ± 0.00
AFL	81.30 ± 11.10	NA	809.2 ± 20.00	890.5 ± 0.18	0.09 ± 0.01	NA
LI (1–2)	72.48 ± 10.50	NA	798.42 ± 25.66	870.9 ± 27.73	0.08 ± 0.01	NA

*Note:* $\sigma_{a}^{2}$ = direct additive genetic variance; $\sigma_{\mathrm{pe}}^{2}$ = permanent environmental variance; $\sigma_{e}^{2}$ = residual variance; $\sigma_{p}^{2}$ = phenotypic variance = $\sigma_{a}^{2}$ + $\sigma_{\mathrm{pe}}^{2}$ + $\sigma_{e}^{2}$; *h* ^2^ = heritability = $\sigma_{a}^{2}/\sigma_{p}^{2}$: *R* = Repeatability = ($\sigma_{a}^{2}$ + $\sigma_{\mathrm{pe}}^{2}$)/$\sigma_{p}^{2}$. Estimates are presented with their standard errors (SE). SE values smaller than 0.005 were rounded to 0.00 at two decimals.

### 3.2 Genetic Trends

**FIGURE 1:**
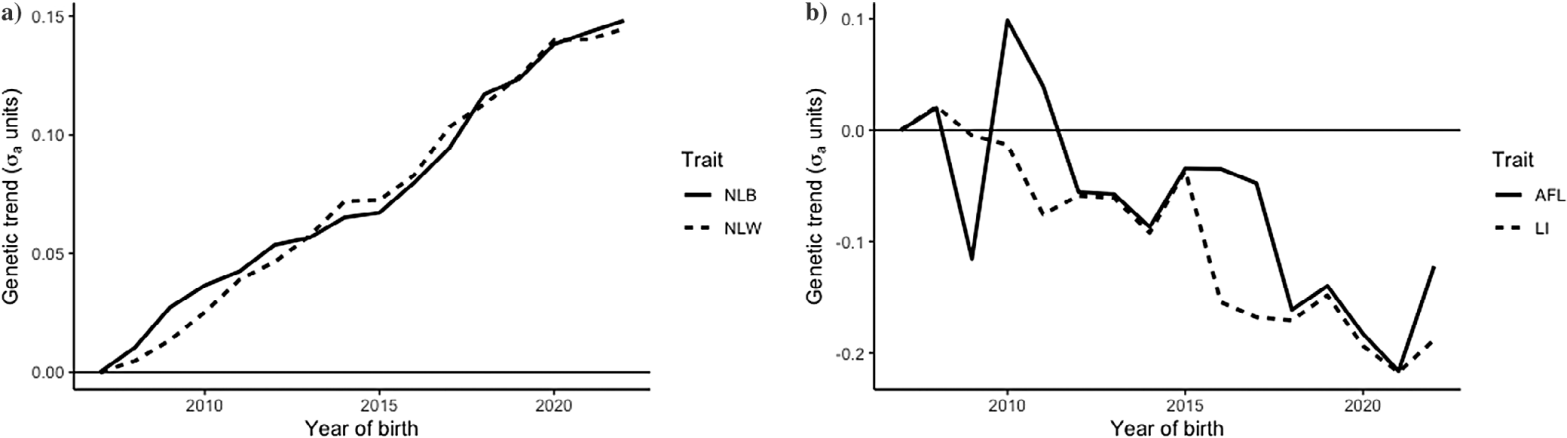
Genetic trends scaled by the additive genetic standard deviation ($\hat{\sigma_{a}}$) for reproductive traits in Katahdin sheep: (a) number of lambs born (NLB) and weaned (NLW); and, (b) age at first lambing (AFL) and interval between first and second lambings (LI).

The estimated genetic trends for NLB and NLW by birth year, from 2007 to 2023, were based on females with reproductive records and are presented relative to animals born in 2007, which was used as the genetic base year. Both traits showed a positive genetic trend over time, indicating accumulated genetic progress in the population (Figure 1a). Figure 1b presents the estimated genetic trends for AFL and LI. Although these traits are not included in the NSIP genetic evaluations and have not been directly targeted for selection, they appear to have been indirectly affected by selection on correlated traits. The genetic trend for AFL showed a fluctuating pattern over time, whereas LI exhibited a more pronounced decline beginning around 2016.

### 3.3 GWAS and Gene Annotation Analyses

**FIGURE 2:**
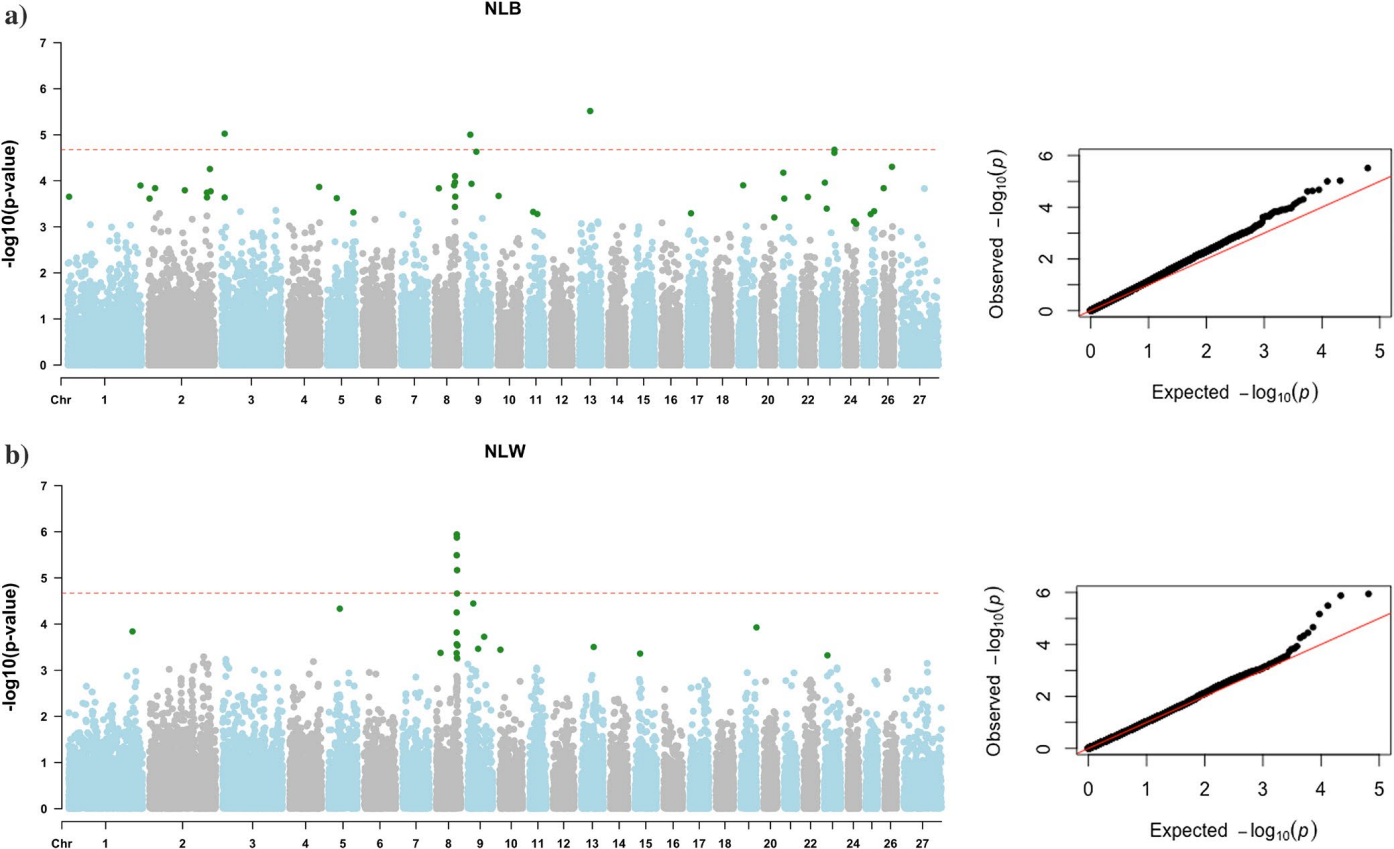
Manhattan plots showing the −log10(*p*‐values) for the association of single nucleotide polymorphisms (SNPs) (left side) and quantile‐quantile plots (QQ‐plot) for (a) number of lambs born (NLB) with genomic inflation factor (*λ*) of 1.00, and (b) for the number of lambs weaned (NLW) with *λ* also equal to 1.00. The significant SNP at the chromosome‐wise threshold are highlighted in green. The horizontal red line indicates the genome‐wise significance threshold. [Colour figure can be viewed at wileyonlinelibrary.com]

**FIGURE 3:**
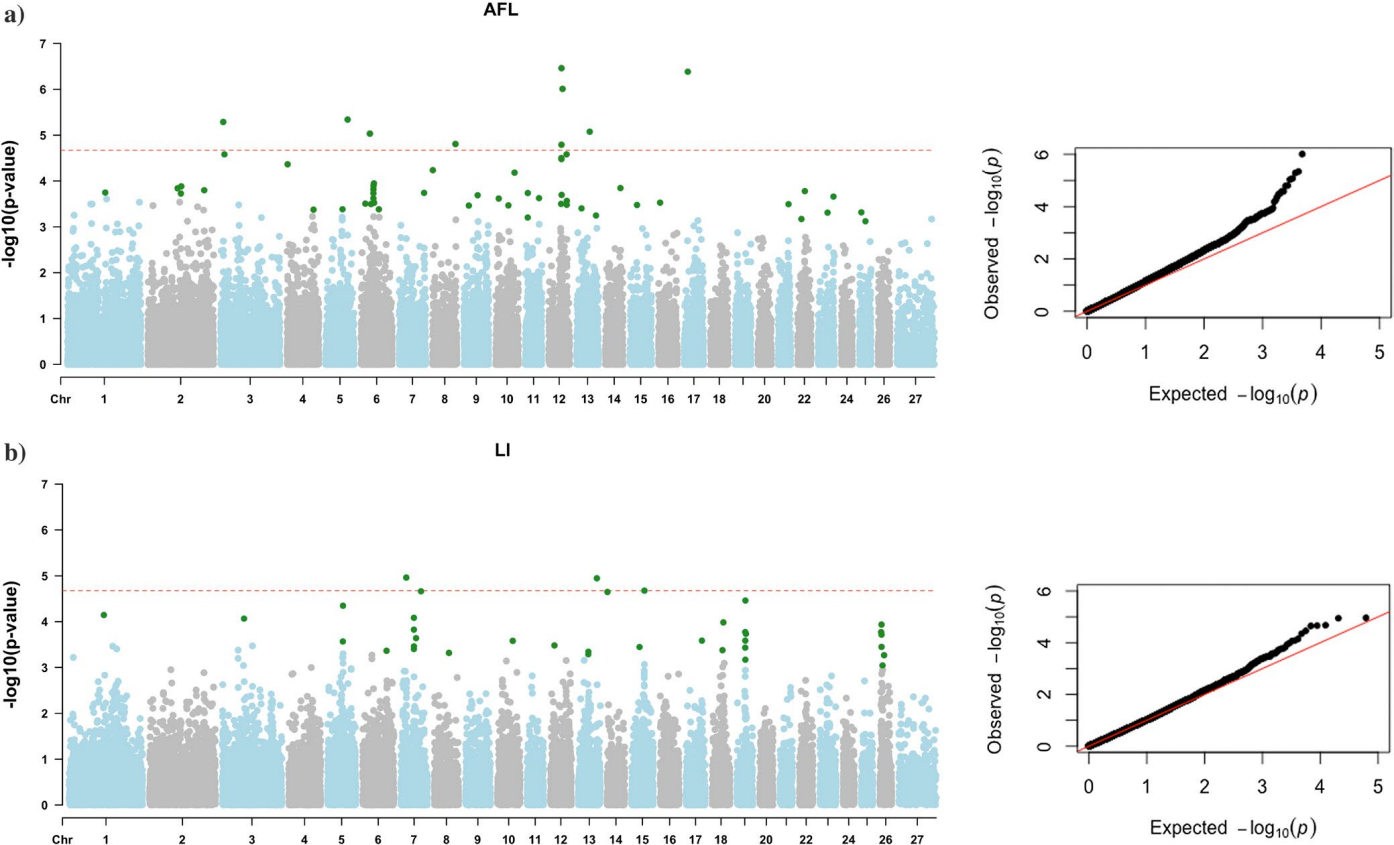
Manhattan plots showing the −log10(*p*‐values) for the association of single nucleotide polymorphisms (SNPs) (left side) and quantile‐quantile plots (QQ‐plot) for (a) age at first lambing (AFL) with genomic inflation factor (*λ*) of 1.00, and (b) interval from first to second lambing (LI) with *λ* equal to 1.03. The significant SNP at the chromosome‐wide threshold are highlighted in green. The horizontal red line indicates the significance of the genome‐wide threshold. [Colour figure can be viewed at wileyonlinelibrary.com]

The Manhattan plots for the NLB, NLW, AFL, and LI traits are presented in Figures 2 and 3. Fourteen significant SNPs were identified and nine suggestive SNPs for the four traits evaluated, which were associated with 26 candidate genes (Table 3). Suggestive SNP were included as exploratory signals to highlight genomic regions of potential interest in traits with complex genetic architecture and low heritability estimates. The complete list of SNPs, their annotations, and the corresponding candidate genes are presented in Table 3. In addition, 22 previously reported QTL were identified within ±100 kb around the significant SNPs: six for NLB, three for NLW, eight for AFL, and five for LI (Table 4).

**TABLE 3:** Significant SNP regions and associated candidate genes for reproductive traits in Katahdin sheep.

Trait	SNP	*p*	OAR	Position	Gene symbol	MAF	Effect
Number of lambs born (NLB)	rs41590104	8 × 10^−06^	3	38,982,368	*AAK1, NFU1, ENSOARG00020034341, GFPT1*	0.45	1 × 10^−04^
	rs59038766	7 × 10^−04^	8	55,364,604	*ARHGAP18, TMEM244, ENSOARG00020033616*	0.46	7 × 10^−05^
	rs07385	8 × 10^−05^	9	50,543,399	*GDAP1, JPH1, ENSOARG00020034674*	0.11	−1 × 10^−04^
	rs29805608	6 × 10^−05^	23	28,612,422	*ENSOARG00020028697*	0.43	−9 × 10^−05^
		6 × 10^−05^	13	30,216,481	*AQP4, SLC23A2*	0.43	−9 × 10^−05^
Number of lambs weaned (NLW)	rs59038766	7 × 10^−05^	8	55,364,604	*ARHGAP18*	0.45	2 × 10^−05^
	rs88609799	6 × 10^−05^	8	89,684,335	*TTLL2, ENSOARG00020040467, ENSOARG00020036644, UNC93A, CCR6, GPR31*	0.14	1 × 10^−05^
	rs82161543	6.03 × 10^−05^	8	30,826,253	*CRYBG1*	0.12	−1 × 10^−04^
	rs75543435	7 × 10^−05^	8	76,726,988	*ESR1*	0.28	−8 × 10^−05^
Age at first lambing (AFL)	rs21972487	4 × 10^−05^	13	22,291,343	*DNAJC1, MLLT10*	0.48	−1 × 10^−05^
	rs35195533	2 × 10^−06^	12	36,321,399	*CCDC181, SLC19A2, NME7*	0.42	3 × 10^−05^
	rs35195425	1 × 10^−05^	12	36,321,291	*BLZF1*	0.41	4 × 10^−05^
	rs106104383	3 × 10^−06^	6	107,183,414	*HS3ST1, ENSOARG00020031336*	0.33	−5 × 10^−05^
	rs88211779	2 × 10^−05^	3	88,287,596	*ENSOARG00020001564*	0.18	−1 × 10^−05^
	rs40537311	4 × 10^−05^	6	41,249,513	*KCNIP4*	0.05	1 × 10^−04^
	rs35937547	4 × 10^−05^	6	36,650,226	*NAP1L5, HERC3, FAM13A*	0.04	−6 × 10^−05^
	rs02537.1	2 × 10^−04^	5	7,746,433	*NOTCH3, ENSOARG00020003710*	0.04	−6 × 10^−06^
Lambing interval (LI)	rs64479	3 × 10^−05^	13	54,901,352	*TAF4, CDH4, ENSOARG00020038098*	0.33	−3 × 10^−05^
	rs66122063	4 × 10^−04^	15	9,660,512	*CNTN5*	0.43	2 × 10^−04^
	rs02561.1	5 × 10^−04^	15	25,397,600	*CADM1*	0.22	−5 × 10^−04^
	rs105704641	6 × 10^−04^	5	10,888,703	*KLF1, DNASE2, ENSOARG00020030506*	0.17	−3 × 10^−05^
	rs31647698	9 × 10^−04^	7	91,046,306	*SEL1L*	0.39	−2 × 10^−04^
	rs22257932	1 × 10^−03^	19	16,699,820	*ENSOARG00020037787, CRELD1*	0.31	−1 × 10^−04^

**TABLE 4:** Significant quantitative trait loci (QTL) regions and associated candidate genes for reproductive traits in Katahdin sheep.

Trait	SNP	QTL ID	Chr	Start Pos	End Pos	*p*	MAF	Gene symbol
Number of lambs born (NLB)	rs22492081	130,458	13	57,353,098	57,369,710	2 × 10^−05^	0.47	*NPEPL1*
	rs16308358	169,628	9	15,620,264	15,652,144	4 × 10^−05^	0.24	*SLC45A4*
	rs16308358	169,628	9	15,653,877	15,709,984	4 × 10^−05^	0.24	*DENND3*
	rs159321398	213,306	3	179,421,566	179,438,606	8 × 10^−05^	0.39	*MB*
	rs76604248	130,458	13	57,381,262	57,407,443	4 × 10^−05^	0.47	*STX16*
	rs27396738	169,188	13	69,972,841	69,979,028	4 × 10^−05^	0.21	*EMILIN3*
Number of lambs weaned (NLW)	rs59038766	211,610	8	50,160,739	50,160,743	2 × 10^−05^	0.46	*CGA*
	rs88609799	211,620	8	50,258,320	50,258,324	4 × 10^−06^	0.26	*HTR1E*
	rs25841996	140,256	8	23,627,047	23,627,051	4 × 10^−05^	0.35	*HS3ST5*
Age at first lambing (AFL)	rs02537.1	300,924	5	10,703,304	10,742,508	1 × 10^−05^	0.34	*IER2*
	rs98426109	300,924	5	10,778,868	10,785,795	6 × 10^−04^	0.34	*LYL1*
	rs02537.1	300,924	5	10,748,416	10,767,067	8 × 10^−05^	0.34	*NACC1*
	rs98426109	300,924	5	10,787,359	10,881,278	2 × 10^−03^	0.34	*NFIX*
	rs40537311	170,229	6	115,201,826	115,266,328	4 × 10^−05^	0.03	*SH3TC1*
	rs98426109	300,924	5	10,766,295	10,777,179	7 × 10^−04^	0.34	*TRMT1*
	rs35937547	288,215	6	33,076,915	33,077,021	3 × 10^−03^	0.40	*U6*
	rs35195533	137,084	12	66,444,926	66,444,930	3 × 10^−05^	0.42	*HMCN1*
Lambing interval (LI)	rs64479.1	154,680	13	23,706,809	23,751,736	1 × 10^−04^	0.25	*AMFR*
	rs02561.1	255,285	15	37,261,282	37,265,085	2 × 10^−04^	0.17	*CALCA*
	rs14211767	259,459	15	15,698,360	15,855,334	5 × 10^−04^	0.43	*CWF19L2*
	rs73512.1	154,680	13	23,685,599	23,702,406	2 × 10^−04^	0.21	*NUDT21*
	rs59582252	239,955	15	47,856,739	47,856,860	9 × 10^−04^	0.01	*U1*

Abbreviations: Chr, chromosome number; MAF, minor allele frequency; Pos, position on the genome; SNP, SNP identification.

#### 3.3.1 Number of Lambs Born (NLB)

For NLB (Table 3), the *AAK1* gene (AP2 associated kinase 1), near a significant SNP (rs41590104) located on OAR3:38,982,368, encodes functions related to protein phosphorylation (GO:0006468) and the regulation of clathrin‐dependent endocytosis (GO:2000369). Another gene, *GFPT1* (glutamine‐fructose‐6‐phosphate transaminase 1), is involved in the metabolic process of fructose 6‐phosphate (GO:0006002) and the biosynthesis of carbohydrate derivatives (GO:1901137). Additionally, a significant SNP (rs49876546) was found on chromosome OAR13:46,990,962, corresponding to the *SLC23A2* gene (solute carrier family 23 member 2), involved in ascorbic acid metabolism (GO:0019852) and transmembrane transport (GO:0055085) pathways. Two of the most significant QTL on chromosome OAR9 were located near the SNP rs16308358 and were associated with the *SLC45A4* (solute carrier family 45 member 4) and *NPEPL1* (aminopeptidase‐like 1) genes (Table 4).

#### 3.3.2 Number of Lambs Weaned (NLW)

Three significant genomic regions associated with the NLW trait were identified (Table 3). One of them was located at SNP rs59038766 on chromosome OAR8:55,364,604, associated with the *ARHGAP18* gene (Rho GTPase activating protein 18), which is involved in signal transduction processes (GO:0007165) and regulation of actin filament polymerisation (GO:0030833). Another significant region was identified at SNP rs88609799 on OAR8:89,684,335, which was annotated to the *TTLL2* (tubulin tyrosine ligase like 2) and *UNC93A* (unc‐93 homolog A) genes. The *TTLL2* gene is involved in microtubule cytoskeleton organization (GO:0000226) and protein polyglutamylation (GO:0018095), whereas *UNC93A*, also located in this region, is related to protein polyglutamylation (GO:0018095). Two of the most significant QTL on OAR8 were also found near SNP rs59038766 and were associated with the *CGA* (glycoprotein hormones, alpha polypeptide) and *HTR1E* (5‐hydroxytryptamine receptor 1E) genes.

#### 3.3.3 Age at First Lambing (AFL)

For AFL, five genomic regions with significant SNPs were identified (Table 3). One such region included the SNP rs35937547 on OAR6:36,650,226, associated with *NAP1L5* (nucleosome assembly protein one like 5), which is involved in nucleosome assembly (GO:0006334), and *FAM13A* (family with sequence similarity 13 member A). Another significant region was identified around the SNP rs106104383 at OAR6:107,183,414, associated with the *HS3ST1* gene (heparan sulfate‐glucosamine 3‐sulfotransferase 1), whose molecular function includes sulfotransferase activity on glucosamine from heparan sulfate (GO:0008467). Three additional significant QTL were also identified: one at SNP rs35195533 at OAR12:66,444,926, associated with the *HMCN1* (Hemicentin 1) gene; another at SNP s02537.1 on chromosome OAR5, associated with the *NACC1* (nucleus accumbens associated 1) gene.

#### 3.3.4 Lambing Interval (LI)

Two significant genomic regions were identified for LI. One of them corresponds to SNP rs64479.1 on OAR13:54,901,352, associated with *TAF4* (TATA‐box binding protein associated factor 4), related to DNA‐directed transcription (GO:0006351) and *CDH4* (cadherin 4), involved in the development of multicellular organisms (GO:0007275), cadherin‐mediated cell–cell adhesion (GO:0044331), and cell morphogenesis (GO:0000902).

The second region was represented by SNP rs31647698.1 located on chromosome OAR7:91,046,306, near the *SEL1L* (SEL1L adaptor subunit of SYVN1 ubiquitin ligase) gene, which is related to the endoplasmic reticulum‐associated degradation (ERAD) pathway (GO:0036503). Additionally, three QTL were identified on OAR15 (Table 4). The SNP rs02561.1 was also associated with the *CALCA* (calcitonin‐related polypeptide alpha) gene. Similarly, the SNP rs64479.1 in OAR13 was associated with the *AMFR* (autocrine motility factor receptor) gene.

## 4 Discussion

### 4.1 Variance Components

The Katahdin breed is characterised by their reproductive and maternal trait performance. Therefore, the breed represents a relevant population for evaluating genetic parameters for traits related to fertility and litter size, such as NLB, NLW, AFL, and LI. We estimated variance components and analysed GWAS signals for these traits in Katahdin sheep to understand their genetic background and identify candidate genes and QTL associated with them. We also incorporated gene ontology data to better understand the biological processes involved in their phenotypic expression.

For all the traits (Table 2), the residual variance ($\sigma_{e}^{2}$) accounted for the largest proportion of the total phenotypic variance, reflecting environmental effects together with other sources of variation not explicitly modelled (e.g., non‐additive genetic effects), as commonly observed for reproductive traits (Ma et al. 2019; Ajafar et al. 2022). The heritability estimates for NLB and NLW were similar to those previously reported in Katahdin sheep using pedigree and phenotypic information alone, with values of 0.09 ± 0.01 and 0.06 ± 0.01 (Notter et al. 2018). Studies on reproductive traits in this breed remain limited, and this is the first study to apply the ssGBLUP methodology for analysing these traits. The heritability estimate for NLB also was comparable to estimates reported for other sheep breeds, such as Rambouillet (0.09 ± 0.01; Araujo et al. 2023), Merino (0.04 ± 0.00; Bolormaa et al. 2017), and crossbred dairy sheep (0.07 ± 0.02; Murphy et al. 2017). Similarly, the heritability estimate for NLW was comparable to that reported for the Dorper breed (0.08 ± 0.01; Zishiri et al. 2013). Although heritability estimates were low, their magnitude is within the range previously reported for other sheep populations. Genetic improvement of these traits is likely to greatly benefit from genomic selection for obtaining more accurate breeding values for selection candidates at younger ages.

The genetic trends observed for NLB (Figure 1) showed clear genetic progress over time, with a sustained increase in the average NLB. In the NSIP Maternal Hair Index, NLB is given a slightly negative weight, while positive emphasis is placed on NLW and growth traits. The Katahdin Hair Sheep Breed Association has also recommended that producers opt for selecting animals with intermediate breeding values for NLB and emphasising twinning to balance productivity and management (https://katahdins.org/using‐ebvs‐to‐select‐replacements‐kathy‐bielek‐ohio‐roxanne‐newton‐georgia). This could explain the more moderate pace of genetic improvement for the NLB trait observed in recent years. Furthermore, the apparent slowdown in recent genetic trends for NLB is consistent with previous studies indicating that, under genomic selection, observed genetic trends in recent generations may level off due to preselection bias rather than a true lack of genetic progress (Masuda et al. 2018).

A similar genetic trend was observed for NLW, with steady improvements since 2017, suggesting increased prolificacy and improved lamb survival at weaning. According to NSIP guidelines, it is generally more effective to select animals with high EBV values for NLW rather than NLB, as NLW reflects both the ewe's reproductive capacity and maternal performance. Taken together, these trends demonstrate significant genetic progress in both the NLB and the ability of ewes to rear their offspring, which is crucial for improving long‐term flock productivity.

Heritability estimates for AFL and LI were 0.09 ± 0.01 and 0.08 ± 0.01, respectively, which are similar to those obtained for NLB and NLW. These results are consistent with previously reported heritabilities for reproductive traits. The low values are expected considering the strong environmental influence on these traits, particularly pronounced in sheep as production systems are highly diverse in management, nutrition, and reproductive practices. Although no previous estimates were found for these traits in the Katahdin breed, comparable values have been reported in other sheep breeds. For AFL, slightly higher heritability estimates have been reported in the Moghani breed (0.10 ± 0.01; Jafaroghli et al. 2022), in Churra Española dairy sheep (0.13 ± 0.04; Vanimisetti and Notter 2012), and in the Lori‐Bakhtiari breed (0.07 ± 0.01; Abdoli et al. 2019a, 2019b).

For the LI trait, even lower estimates than those obtained in the current study have been reported in other sheep populations, such as in Santa Inés (0.04 ± 0.01; Oliveira et al. 2024), Lori‐Bakhtiari (0.02 ± 0.00; Abdoli et al. 2019a, 2019b), and Dorper (0.01 ± 0.00; Zishiri et al. 2013) breeds. Despite the low heritability, the observed genetic trends (Figure 1) suggest that genetic progress has been obtained for these traits. AFL and LI are not part of the official NSIP selection indexes and have not been directly selected for. AFL showed marked interannual fluctuations, with an overall slight decreasing genetic trend over time, consistent with its low heritability.

In contrast, LI showed a downward trend since 2016, suggesting potential indirect genetic improvement. These traits are relevant to herd productivity and may serve as indicators of ewe longevity. According to Vanimisetti and Notter (2012) and Pinto et al. (2024), younger AFL is associated with a longer productive lifespan, which is essential for the sustainability of the production system. Therefore, incorporating AFL and LI into routine NSIP genetic evaluations could provide additional value by improving the population's reproductive efficiency and longevity.

The estimated genetic correlation of 0.79 between NLB and NLW is consistent with previous studies in the Katahdin breed, which reported similar or slightly higher values, such as 0.70 (Vanimisetti et al. 2007) and 0.88 (Notter et al. 2018). Similarly, in research on Rideau‐Arcott sheep, Nel (2022) reported a genetic correlation of 0.67 between NLB and NLW. Although still positive, the genetic correlation between AFL and LI was much smaller (0.17). Although no specific estimates have been reported for this correlation in Katahdin sheep, similar values have been observed in other breeds, such as 0.10 in Bonga sheep (Tera et al. 2021) and 0.19 in a multi‐breed sheep population (Lôbo et al. 2009). These results suggest a weak but potentially relevant genetic relationship between these two reproductive traits, which could be useful in integrated selection strategies. Overall, our results indicate that genetic progress is achievable over time, even for traits that are lowly heritable such as NLB and NLW. The observed genetic trends and the high genetic correlation between these traits reflect both the direct and correlated responses to selection in the Katahdin breeding program.

### 4.2 Genome‐Wide Association Studies and Functional Analyses

#### 4.2.1 Number of Lambs Born (NLB)

Although no previous study reported associations of *AAK1* with reproductive traits in sheep, this gene has been linked to hair follicle development and UV radiation protection in Cashmere goats (Wu et al. 2023) as well as to environmental adaptation in Hainan Yellow cattle (Zhong et al. 2024). These functions suggest a potential role for *AAK1* in physiological adaptation and tissue development, both relevant to reproductive efficiency.

The *GFPT1* gene, was previously associated with fat deposition and body mass index in Lori‐Bakhtiari sheep (Ghiasi and Abdollahi‐Arpanahi 2021). This association suggests that the *GFPT1* gene may influence female body condition and energy reserves, which in turn could support greater reproductive output (e.g., larger litter size; Ghiasi and Abdollahi‐Arpanahi 2021). The *SLC23A2* gene has been reported in Barbarine sheep that are more adapted to thermal and nutritional stress (Yahyaoui et al. 2024), suggesting an indirect contribution to reproductive performance through improved resilience under adverse environmental conditions. *SLC23A2* has also been linked to thermotolerance in African cattle (Taye et al. 2017) and with IGF (insulin‐like growth factor)‐mediated signalling pathways in Landrace pigs (Jung et al. 2024), which are involved in follicular development and ovulation. These functions are relevant for productive performance in challenging environments, particularly through their impact on cellular energy balance and stress responses.

The *NPEPL1* gene has been reported in birds of the Xinyang Blue breed in China (Hou et al. 2020), suggesting that it may influence fertility and prolificacy through hormonal and enzymatic pathways. Additionally, *SLC45A4*, has been implicated in glycogen synthesis at the onset of lactation in rats (Patel et al. 2019). These genes collectively support a potential role in both reproductive output (e.g., litter size in sheep) and metabolic readiness for lactation.

#### 4.2.2 Number of Lambs Weaned (NLW)

For NLW, several significant regions were identified on OAR8 (Table 3). Among them, the *ARHGAP18* gene has been associated with ovarian steroidogenesis and follicular development in goats (Liu, Feng, et al. 2024; Liu, Liu, and Chu 2024). In Nellore cattle, this gene has been linked to maintaining endothelial cell alignment, and altered gene function has been associated with negative effects on longevity (Ogunbawo et al. 2025). The *ARHGAP18* gene was also associated with NLB, which is particularly relevant because NLB and NLW are genetically correlated, implying that shared biological mechanisms could regulate both traits. Although specific studies on *ARHGAP18* in sheep are lacking, research in other species, such as goats and cattle, has linked this gene to key reproductive processes, including ovarian steroidogenesis, follicular development, and endothelial cell integrity, all of which are closely related to fertility and reproductive longevity.

The *TTLL2* gene is related to processes in cell division, intracellular transport, and cell signalling (Tang et al. 2019). Similarly, *UNC93A* is involved in synaptic activity and regulation of membrane trafficking, though its specific function in livestock is not yet fully understood (Ceder et al. 2017). In addition, QTL detected on OAR8 (Table 4) are associated with the *CGA* gene, which has been linked to litter size in sheep (Ghiasi and Abdollahi‐Arpanahi 2021), and shows significant annotations related to prolificacy. This gene is involved in gonadotropin‐releasing hormone (GnRH) signalling pathways, which stimulate the release of luteinizing hormone (LH) and follicle‐stimulating hormone (FSH), both essential for ovulation and fertility. The *CGA* gene has been associated with reproductive functions in Pelibuey sheep (Hernández‐Montiel et al. 2020) and with key hormonal regulatory processes in Japanese Black cattle (Abdillah et al. 2022). Given its role in hormonal regulation, the identification of *CGA* in this region suggests a potential functional association with NLW, as it is directly involved in reproductive physiological processes.

QTL associated with the *HTR1E* gene were also identified (Table 4). This gene has been linked to reproductive processes in several sheep breeds. In Pelibuey sheep, *HTR1E* has been associated with the cAMP signalling pathway, which enables persistent hormonal signalling from internalised LH receptors to affect follicular cells and oocyte (Hernández‐Montiel et al. 2020). In Luzhong lambs, *HTR1E* has been implicated in critical hormonal mechanisms regulating reproduction (Tao et al. 2021). Furthermore, *HTR1E* has been specifically associated with NLB (Liu, Feng, et al. 2024; Liu, Liu, and Chu 2024), reinforcing its potential role in traits such as NLB and NLW.

#### 4.2.3 Age at First Lambing (AFL)

For the AFL trait, significant SNP were identified on OAR6, associated with three relevant genes: *NAP1L5*, *FAM13A*, and *HS3ST1* (Table 3). The *NAP1L5* gene has been linked to foetal growth and development in Croatian sheep breeds (Ramljak et al. 2024) and in Hanwoo beef cattle (Naserkheil et al. 2020). Furthermore, according to Jiang et al. (2015), *NAP1L5* is involved in the morula‐to‐blastocyst transition in bovine embryos, suggesting a critical role in early embryonic development. On the same chromosome, *FAM13A* has been associated with reproductive efficiency in Hu sheep (Ma et al. 2024) and with spermatogenesis‐related processes in native sheep (Tomar et al. 2021). It has also been linked to productive traits such as adult weight and body fat distribution in Soay sheep (James et al. 2022).

The *HS3ST1* gene has been associated with immune responses in native Moroccan sheep (Kusza et al. 2024) and with tolerance to hemoparasitic diseases in ruminants (Kalaiyarasi and Elayadeth‐Meethal 2023). In Simmental cattle, this gene has also been linked to backfat thickness (Song et al. 2016), suggesting a potential influence on metabolism and body condition, both of which are relevant to the onset of reproductive maturity. Also, *BLZF1* has been associated with oocyte developmental potential in cattle (Walker and Biase 2020) and with the competence of oocytes from medium‐sized follicles (Nemcova et al. 2016). Together, these genes contribute to biological processes such as embryonic development, sexual maturity, and metabolic readiness, highlighting their potential influence on AFL. These functions highlight the gene's relevance to oocyte quality and reproductive capacity in early life stages.

Several QTL (Table 4) were also located in genomic regions containing genes with important functions in other species, suggesting potential relevance to AFL. On OAR12, a QTL was identified near the *HMCN1* gene, which is involved in cell adhesion and cytoskeletal organization in sheep (Kyselová et al. 2023). This gene has also been associated with residual feed intake in crossbred beef cattle (Abo‐Ismail et al. 2014), suggesting a possible link with energy efficiency and physiological development, both of which are potentially relevant factors for achieving the body development necessary to initiate puberty promptly.

#### 4.2.4 Lambing Interval (LI)

For LI, significant genomic regions associated with four genes located on OAR13, OAR15, and OAR7 were identified (Table 3). On OAR13, the *TAF4* gene has been associated with larger litter sizes in Chuanzhong Black goats (Guo et al. 2024) and with meat yield traits and lean meat ratios in Canadian cattle (Sood et al. 2023), suggesting roles in reproductive efficiency and metabolic function. On OAR7, the *SEL1L* gene was identified, which is expressed in liver tissues with high concentrations of fatty acids in ruminants, particularly in sheep with a high proportion of unsaturated fatty acids (Gunawan et al. 2021). This gene has also been implicated in the growth and differentiation of pancreatic epithelial cells during embryonic development in mice (Li et al. 2010). These functions indicate possible roles in metabolism, hormonal regulation, and reproductive efficiency. Taken together, these genes may influence LI through their involvement in metabolic, immunological, and reproductive pathways.

Several QTL significantly associated with LI (Table 4) also contain genes of functional interest. The *AMFR* gene, located on OAR13, has been previously associated with litter size in sheep (Ghiasi and Abdollahi‐Arpanahi 2021), and with polyunsaturated fatty acid (PUFA) metabolism and milk composition in cattle (Bohlouli et al. 2022). These traits are especially relevant during lactation, a physiological phase that directly impacts the resumption of the reproductive cycle postpartum.

On OAR15, the *CALCA* gene has been associated with reproductive seasonality and the duration of the ovarian cycle in sheep (Martinez‐Royo et al. 2017). Given that LI reflects the time between successive lambings, which depends on the reactivation of the estrous cycle postpartum, the expression of genes like *CALCA*, involved in hormonal and seasonal regulation, may influence this interval. Additionally, its role in embryonic and foetal development in humans (Dong et al. 2007) points to broader reproductive functions. Overall, these genes are associated with reproduction, hormonal regulation, metabolism, and cell viability. Their interactions may affect the timing of postpartum estrus resumption, thereby influencing LI.

### 4.3 Factors Affecting Estimates of Variances and Identification of Genome Wide Associations

Several factors can influence the accuracy of variance component estimates and the identification of genome‐wide associations for complex reproductive traits, including the precision of phenotypic measurements, the number of animals with records per trait, and the statistical models used. In GWAS, biological interpretation is generally limited to genes located near associated markers, as regulatory or distant causal variants may not be captured. Moreover, the GWAS methodology can influence both the number and distribution of detected associations.

The variance components indicate that NLB, NLW, AFL, and LI are significantly influenced by environmental and other non‐modelled (e.g., non‐additive genetic factors) effects; therefore, these factors should be minimised or accounted for to improve future genetic analyses. Genetic progress was observed for NLB and NLW. At the same time, AFL and LI, with similar heritabilities, showed modest improvement, likely reflecting correlated responses to selection on other traits despite not being included in the NSIP index.

In ssGWAS, strong signals were observed for NLB, NLW, and AFL, while fewer significant markers were detected for LI. This pattern is consistent with the low heritabilities estimated for all the studied traits. The reproductive traits studied are polygenic and genetically complex, influenced by multiple factors such as growth, weight gain, immunity, adaptation, and reproductive physiology. These layers of complexity can limit the statistical power to detect associations at the genome‐wide level. Despite the limited number of strong signals, the significant SNPs identified were located near genes and QTL associated with growth, reproduction, adaptation, and disease resistance. These functional annotations are consistent with the biology of the reproductive traits analysed and supported by studies in sheep and other mammals, providing a basis for future research and possible inclusion in breeding programs.

## 5 Conclusions

The reproductive traits evaluated in this study (NLB, NLW, AFL, and LI) are lowly heritable. Nonetheless, all traits have sufficient additive genetic variance to enable genetic improvement through genomic selection. Genetic trends indicated clear genetic progress in NLB and NLW, likely due to their inclusion in the NSIP selection scheme over recent years. Our GWAS enabled the identification of various genomic regions and candidate genes associated with reproductive performance in Katahdin sheep. These genes play key roles in biological pathways related to fertility, prolificacy, precocity, growth, follicular and embryonic development, disease resistance, and environmental adaptation. Given the polygenic nature and slow albeit steady genetic progress observed for the studied reproductive traits, incorporating additional or composite reproductive indicators into breeding programs like NSIP may enhance selection accuracy and long‐term productivity. Future GWAS efforts using larger datasets, greater SNP density (from genotyping or sequencing sources), and more advanced genomic and biological validation tools will be crucial to validate these findings and further elucidate the genetic architecture of reproduction in Katahdin and other sheep breeds.

## Funding

This study was supported by the National Institute of Food and Agriculture (2022‐67015‐36073 and 2016‐51300‐25723).

## Conflicts of Interest

The authors declare no conflicts of interest.

## Supporting information


**Table S1:** Distribution of the number of records (parities) per ewe for the number of lambs born (NLB) and number of lambs weaned (NLW) in Katahdin sheep.

## Data Availability

The data that support the findings of this study are available from the National Sheep Improvement Program (NSIP). Restrictions apply to the availability of these data, which were used under license for this study. Data are available from the author(s) with the permission of the National Sheep Improvement Program (NSIP).
