# Supplementary material for: Genetic Parameters and Genome‐Wide Association Studies for Fertility and Reproduction Traits in U.S. Katahdin Sheep Based on the Single‐Step GBLUP Methodology
Source: J Anim Breed Genet. 2026 Mar 30;143(5):625–38. doi: 10.1111/jbg.70048 (PMC13460450; doi:10.1111/jbg.70048)
Supplement: Supplementary file 1 [file JBG-143-625-s001.docx]

**SUPPLEMENTARY MATERIAL**

**Table S1.** Distribution of the number of records (parities) per ewe for the number of lambs born (NLB) and number of lambs weaned (NLW) in Katahdin sheep.

| **Number of records (Lambing)** | **Number of ewes** |
| --- | --- |
| 1 | 9,305 |
| 2 | 6,133 |
| 3 | 3,906 |
| 4 | 2,355 |
| 5 | 1,555 |
| 6 | 1,009 |
| ≥7 | 986 |
